# Herpesvirus systematics^☆^

**DOI:** 10.1016/j.vetmic.2010.02.014

**Published:** 2010-06-16

**Authors:** Andrew J. Davison

**Affiliations:** MRC Virology Unit, Institute of Virology, University of Glasgow, Church Street, Glasgow G11 5JR, UK

**Keywords:** Herpesvirus, Classification, Genomics, Herpes simplex virus, Human cytomegalovirus

## Abstract

This paper is about the taxonomy and genomics of herpesviruses. Each theme is presented as a digest of current information flanked by commentaries on past activities and future directions.

The International Committee on Taxonomy of Viruses recently instituted a major update of herpesvirus classification. The former family *Herpesviridae* was elevated to a new order, the *Herpesvirales*, which now accommodates 3 families, 3 subfamilies, 17 genera and 90 species. Future developments will include revisiting the herpesvirus species definition and the criteria used for taxonomic assignment, particularly in regard to the possibilities of classifying the large number of herpesviruses detected only as DNA sequences by polymerase chain reaction.

Nucleotide sequence accessions in primary databases, such as GenBank, consist of the sequences plus annotations of the genetic features. The quality of these accessions is important because they provide a knowledge base that is used widely by the research community. However, updating the accessions to take account of improved knowledge is essentially reserved to the original depositors, and this activity is rarely undertaken. Thus, the primary databases are likely to become antiquated. In contrast, secondary databases are open to curation by experts other than the original depositors, thus increasing the likelihood that they will remain up to date. One of the most promising secondary databases is RefSeq, which aims to furnish the best available annotations for complete genome sequences. Progress in regard to improving the RefSeq herpesvirus accessions is discussed, and insights into particular aspects of herpesvirus genomics arising from this work are reported.

## 1 Introduction

Systematics is usually taken to be synonymous with the classification of organisms, but for the purposes of this paper I have employed a broader definition that includes both taxonomy and the systematic aspects of genomics. I address aspects of how the current situation in each area has been reached and how it might develop in future, and provide detailed summaries of current information. I also discuss a selection of new findings that have emerged from genomic studies. Readers should note that herpesvirus names are referred to in this paper not in full but by the acronyms listed in Table 1.

## 2 Herpesvirus taxonomy

### 2.1 Introduction

Taxonomy – the systematic classification of living organisms – is an exercise in categorization that helps human beings cope with the world. Of the taxa into which organisms may be classified, only those of species, genus, subfamily, family and order are presently applicable to viruses. The grouping of viruses into these taxa proceeds on the basis of identifying shared properties, and has applications in understanding virus evolution, identifying the origins of emerging diseases, and developing rational treatment strategies.

The criteria used to classify viruses have necessarily depended on the knowledge and technology available at the time. In early days, aspects of biology such as host range and broad pathogenic and epidemiological features of the disease constituted the majority of information available. With the advent of electron microscopy and physicochemical methods, data on the morphological properties and constituents of virus particles became accessible, and these are still important today in assigning viruses to higher taxa. Thus, for example, all herpesviruses share a capsid structure that consists of a DNA core surrounded by an icosahedral (20-faceted) capsid consisting of 12 pentavalent and 150 hexavalent capsomeres. The capsid is embedded in a proteinaceous matrix called the tegument, which in turn is invested in a glycoprotein-containing lipid envelope. The advent of specific antibodies facilitated the study of antigenic relationships, which are generally detectable only between closely related viruses; in the case of herpesviruses, those in the same genus. The era of nucleic acid sequencing has led to the wide application of sequence-based phylogeny to classification. Indeed, the quantitative basis and broad utility of this approach have resulted in this becoming a key taxonomical discriminator in all parts of the tree of life, to the point where it dominates all other criteria.

### 2.2 Resources

Advances in virus taxonomy have been recorded in a series of eight reports published at intervals since 1971 by the International Committee on Taxonomy of Viruses (ICTV). The latest report in book form was published in 2005 (Fauquet et al., 2005). In deference to the electronic age, annual updates of the list of virus species were published in 2007, 2008 and 2009 at the ICTV website (http://www.ictvonline.org).

Virus classification is advanced through a voting procedure involving the members of the ICTV. The proposals on which voting takes place are prepared by the ICTV Executive Committee from submissions made by individuals in the virological community, in particular those associated with Study Groups devoted to particular virus groups (usually families). The development of taxonomy as summarized in the ICTV reports is regularly promoted, supplemented and discussed by expert publications from Study Groups, including that focused on the herpesviruses.

### 2.3 Past

In the first ICTV report (Wildy, 1971), the genus *Herpesvirus* was established, consisting of 23 viruses and 4 groups of viruses named according to the usages of the day (e.g. herpesvirus of saimiri). In the second ICTV report (Fenner, 1976), this genus was elevated to the family *Herpetoviridae*, which, presumably because of the misleading association of this name with reptiles and amphibians, was renamed *Herpesviridae* in the third ICTV report (Matthews, 1979). Also, a formal system for naming herpesviruses was founded (Roizman et al., 1973), implemented (Fenner, 1976; Matthews, 1979), elaborated (Roizman et al., 1981) and consolidated (Francki et al., 1991; Roizman et al., 1992).

This naming system specified that each herpesvirus should be named after the taxon (family or subfamily) to which its primary natural host belongs. The subfamily name was used for viruses from members of the family Bovidae or from primates (the virus name ending in –ine, e.g. bovine), and the host family name for other viruses (ending in –id, e.g. equid). Human herpesviruses were treated as an exception (i.e. human rather than hominid). Following the host-derived term, the word herpesvirus was added, succeeded by an arabic number, which bore no implied meaning about the taxonomic or biological properties of the virus. Thus, the formal name of pseudorabies virus (also known as Aujeszky's disease virus) was established as suid herpesvirus 1. Since herpesviruses had previously been named on an *ad hoc* basis, sometimes with the effect that a virus might have several names, the formal system promised a degree of clarity and simplicity to students and scientists in the research field. However, a number of practical disadvantages of the formal naming system emerged. Most importantly, many virus names (e.g. Epstein–Barr virus) were so widely accepted that they could not be dislodged (e.g. in this case by human herpesvirus 4). This led to the use of a dual nomenclature in the literature for some herpesviruses.

Nonetheless, classification of herpesviruses continued and expanded. At the time of the third ICTV report (Matthews, 1979), the family *Herpesviridae* was divided into 3 subfamilies (*Alphaherpesvirinae*, *Betaherpesvirinae* and *Gammaherpesvirinae*) and 5 unnamed genera, and 21 viruses were listed. A subsequent list compiled by the Study Group contained 89 viruses (Roizman et al., 1981).

At the time of the seventh report (Minson et al., 2000), the ICTV adopted the species concept, which recognizes that a virus and the species to which it belongs fall into different categories, the real and the conceptual (Van Regenmortel, 1990). This sea change put the activities of the ICTV on a more logical footing, and also limited its authority to determining formal taxonomical nomenclature and classification, and not virus names, abbreviations and vernacular usages of formal names. It also brought about a simplification by effectively removing any implied taxonomical standing for viruses denoted previously as tentative species or unassigned viruses. Moreover, it reduced tensions concerning the pervasive use of a dual system for herpesvirus names, with the effect that the ICTV approach has been adopted for some (e.g. equine abortion virus is now well known as equid herpesvirus 1) and the *ad hoc* name for others (e.g. Kaposi's sarcoma-associated herpesvirus, rather than human herpesvirus 8). The rules for virus taxonomical names are that they are written in italics with the first letter capitalized, and never abbreviated. Thus, for example, pseudorabies virus belongs to the species *Suid herpesvirus 1*, genus *Varicellovirus*, subfamily *Alphaherpesvirinae*, family *Herpesviridae*. If an abbreviation based on the formal name is used (in this case, SuHV1 or SuHV-1), it represents the virus and not the species.

As the classification developed, it became clear from genome studies that IcHV1 and OsHV1 are very distant relatives of each other and other herpesviruses. For this reason, a new order, *Herpesvirales*, was created (Davison et al., 2009). This accommodates three families, namely the revised family *Herpesviridae*, which contains mammal, bird and reptile viruses, the new family *Alloherpesviridae*, which incorporates bony fish and frog viruses, and the new family *Malacoherpesviridae*, which contains OsHV1. Also, species representing viruses of non-human primates were renamed after the host genus rather than the subfamily, in order to cope with their rising number.

### 2.4 Present

The most recent update (2009) of herpesvirus taxonomy largely concerned the introduction of additional taxa to the family *Alloherpesviridae*. The current, complete list of herpesvirus taxa is given in the first column of Table 1. This table also provides the common names and acronyms (abbreviations) of the viruses. The right-hand part of the table conveys genomic information, which is discussed below. The order *Herpesvirales* now consists of 3 families, 3 subfamilies, 17 genera and 90 species. A total of 48 tentative species and unassigned viruses are also listed in Table 1, but, as discussed above, these viruses are not part of formal taxonomy and are included out of convenience rather than necessity. Many are longstanding entities that may no longer exist in laboratories and for which sequence data are not (and never will be) available. Moreover, some are now known or suspected to be strains of other viruses (see the footnotes to Table 1).

### 2.5 Future

The ICTV *Herpesvirales* Study Group is continuing to progress the classification of herpesviruses as they are identified and characterized. It is also taking forward discussions that will shape herpesvirus taxonomy in future. These include revisiting the herpesvirus species definition and the criteria used for taxonomic assignment, particularly in regard to the possibilities of classifying the large number of herpesviruses detected only as DNA sequences by polymerase chain reaction (PCR).

The ICTV follows the principle that “a virus species is a polythetic class of viruses that constitutes a replicating lineage and occupies a particular ecological niche” (Van Regenmortel, 1992). The polythetic nature of virus species (that is, having some but not all properties in common) implies that a species cannot be delineated on the basis of a single property. In line with this principle, herpesviruses are classified as distinct species if “(a) their nucleotide sequences differ in a readily assayable and distinctive manner across the entire genome and (b) they occupy different ecological niches by virtue of their distinct epidemiology and pathogenesis or their distinct natural hosts” (Roizman et al., 1992; Minson et al., 2000; Davison et al., 2005a). However, the dominant criterion in virus classification, as in the classification of all organisms, is now sequence-based phylogeny. The impact of this development upon the continuing viability of herpesvirus classification under the polythetic rule will need careful consideration. Discussions are likely to be driven to some extent by the ongoing PCR-based discovery of large numbers of herpesviruses that potentially belong to new taxa (e.g. Ehlers et al., 2008). Finally, the nomenclature of herpesvirus species is inherently unstable, because of its dependence on host nomenclature and the motivation to rename species that become very numerous in certain host families. It will be a challenge to maintain a balance between utility and stability.

## 3 Herpesvirus genomics

### 3.1 Introduction

Genomics – the study of the structure, function and evolution of genomes – underlies most discoveries in modern biology. Since the inception of nucleic acid sequencing, and increasingly since, interpretation of the data has lagged behind its generation. Hence, understanding the meaning of genomic data is at a premium. Interpretation of sequence data is served greatly by computer analyses in areas such as comparative genomics, pattern-based bioinformatics and proteomics. However, it is not best advanced when treated as a robotic exercise. Genomics, even of herpesviruses, has an actively speculative edge where new discoveries are being made and old models are being refined.

### 3.2 Resources

The primary nucleotide sequence databases, of which perhaps the most prominent is GenBank at the National Center for Biotechnology Information (NCBI; http://www.ncbi.nlm.nih.gov), are a vital resource to experimenters. NCBI is also putting effort into the Reference Sequence Project (RefSeq; http://www.ncbi.nlm.nih.gov/RefSeq), which aims to provide a comprehensive, integrated, non-redundant, well annotated set of sequences for major research organisms, including viruses (http://www.ncbi.nlm.nih.gov/genomes/GenomesHome.cgi?taxid=10239) (Pruitt et al., 2003). The herpesvirus RefSeqs are under development (http://www.ncbi.nlm.nih.gov/genomes/GenomesGroup.cgi?taxid=548681).

### 3.3 Past

The utility of the primary databases is limited by three major factors: the accuracy of the sequence, the quality of the annotation (i.e. the description of features in the genome—genes, RNAs, coding regions and so forth), and the improvements made to both over time. However, for understandable reasons, updating an accession is effectively restricted to the original depositor. The tendency is for this to be done rarely, if at all, and this is causing the primary databases to become antiquated. Against this background, an enterprise in updating herpesvirus accessions in primary sequence databases seems forlorn. RefSeq provides a way to counter the disadvantages of the primary databases by enabling updating and curation by experts other than the original depositor.

### 3.4 Present

The right-hand part of Table 1 lists information on the 51 herpesvirus species or potential species from which at least one virus has been sequenced to date. These species are represented by 122 complete or almost complete genome sequences. Partial sequence information is available on nearly all the other herpesviruses ranked in formal taxa; indeed, this is now a requirement for classification. At an experimental level, many herpesviruses are manipulated in the form of bacterial artificial chromosomes (BACs), the sequences of several of which are available; for example, those representing GaHV2, HHV1 and HHV5. The latest sequencing techniques are starting to be applied to herpesviruses, including the Roche 454 instrument (e.g. GaHV2 and MuHV1) or Illumina Genome Analyzer (e.g. HHV1 and HHV5).

I have been active in updating the herpesvirus RefSeqs, and thus far have processed all members of the subfamily *Alphaherpesvirinae*, plus some other viruses outside this group (e.g. HHV5). In addition to identifying potential sequence errors and upgrading the standard of annotation, one of the main tasks has been to start applying a systematic nomenclature to genome features. Members of the best studied family (*Herpesviridae*) in the order *Herpesvirales* share 44 genes apparently inherited from a common ancestor (core genes; Table 2), and yet the names of these orthologous genes and their encoded proteins vary from virus to virus. This has led to confusion in the research field. My tack has been to retain original gene names so that workers can find their way round an accession, and to apply standard names to the encoded proteins. This is aimed at improving the utility of the database accessions; for example, database searches would then return the same name for orthologous proteins from different viruses. Table 2 shows the current scheme used to annotate the RefSeqs for members of the subfamily *Alphaherpesvirinae*. A more extensive form of this database is available from me in spreadsheet form. As well as providing additional information, this database permits the genes and their encoded proteins to be sorted according to their order along a particular genome.

### 3.5 Future

The long term aim of updating herpesvirus RefSeqs is to ensure that are correct (free from errors), complete (adequately annotated), clear (using standard nomenclature) and current (up to date). From experience, I foresee that fulfilling this aim to an acceptable standard will be demanding and that many considerations will have to be weighed. Most importantly, the list of standard protein names must be considered as needing development in future, since the process of establishing names is fraught with pitfalls that are not developed further here. Thus, as with the names of herpesvirus species in Table 1, the protein names in Table 2 are provisional. It is intended that they will improve and harmonize as knowledge increases and data are imported from the other herpesvirus subfamilies. The substantial efforts of Mocaski (2007) to apply a standard nomenclature to the proteins encoded by core genes also deserve recognition in this regard.

## 4 Interpretations

### 4.1 Introduction

The process of systematizing information as described above tends to yield new insights, particularly those arising from comparative genomics. This section highlights three examples that were unearthed while reannotating genomes of members of the subfamily *Alphaherpesvirinae* and members of the genus *Cytomegalovirus* in the subfamily *Betaherpesvirinae*. These examples illustrate the fact that new discoveries remain to be made even with well characterized herpesviruses.

### 4.2 UL56 gene family

**Fig. 1:**
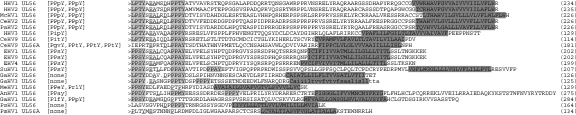
Amino acid sequence alignment of the C-terminal regions of membrane protein UL56 and membrane protein UL56A from members of the subfamily *Alphaherpesvirinae*. The sequences are aligned only in the 15 residues at the left, as relationships among the sequences elsewhere are overall not detectable. The locations of PPXY (and related LPPY) motifs are highlighted in light grey, and other conserved residues located in or between the motifs are underlined. Additional PPXY and PPXY-related motifs in the N-terminal regions (indicated by >) are noted in square brackets. Predicted transmembrane domains are highlighted in dark grey. That for GaHV3 is in lower case to indicate that a correction of a putative frameshift (located approximately) was invoked. The total number of residues in each protein is indicated on the right.

Previously, I reported that orthologues of gene UL56 are not confined to members of the genus *Simplexvirus* in the subfamily *Alphaherpesvirinae*, where they were first identified, but are also found among members of the genera *Varicellovirus* and *Iltovirus* (Davison, 2007). Subsequent analysis (Fig. 1 and Table 2) indicated that orthologues are also present in members of the genus *Mardivirus*. These studies indicate that all members of the subfamily *Alphaherpesvirinae* except BoHV1 and BoHV5 encode a UL56 orthologue. The appropriate amendments were applied to the RefSeqs by the middle of 2007.

All the versions of the UL56 product (membrane protein UL56) have a C-terminal hydrophobic domain, and sequence similarity is limited to a central region consisting in most instances of two PPXY motifs (Fig. 1). Most of the proteins have additional PPXY elements elsewhere in their sequences. Moreover, two viruses (CeHV9 and PsHV1) appear to have additional genes related to UL56, whose products are termed membrane protein UL56A. The existence of these paralogues gives rise to the novel UL56 gene family. This systematic evaluation provides a framework within which to view the possible roles of the encoded proteins.

In cellular proteins, PPXY motifs interact with the WW domain, which is 35–40 residues in length and structured as a 3-stranded, antiparallel β-sheet with two ligand-binding grooves (Macias et al., 1996). The WW domain is invariably joined to one of a wide variety of other protein modules, and is thus implicated in assembly of multiprotein complexes (Ingham et al., 2005). These processes encompass transcription, RNA processing, protein trafficking, receptor signaling, control of the cytoskeleton and vacuolar protein sorting (Hettema et al., 2004). Vacuolar protein sorting is exploited for budding by some enveloped viruses, utilizing PPXY motifs in virus proteins (Wills et al., 1994; Martin-Serrano et al., 2005). These observations from cellular proteins are in general accord with what is known about membrane protein UL56.

In HHV1, UL56 is not required for growth of virus in cell culture. However, mutants, including one lacking the C-terminal hydrophobic domain, are compromised in pathogenicity, including neuroinvasiveness (Peles et al., 1990; Rösen-Wolff et al., 1991; Berkowitz et al., 1994; Kehm et al., 1996), although this appears to depend on the system used (Nash and Spivack, 1994). Moreover, mutants with lesions in UL56 and other genes have been examined for utility in oncolytic viral therapy (Takakuwa et al., 2003; Sugiura et al., 2004; Ushijima et al., 2007). The protein has been detected in virions (Kehm et al., 1994, 1998).

Additional information is available for HHV2, in which UL56 has been shown to encode a type 2 membrane protein that localizes to the Golgi apparatus and cytoplasmic vesicles, and is tail-anchored by the C-terminal hydrophobic domain so that the N terminus (containing the PPXY motifs) is located in the cytoplasm (Koshizuka et al., 2002). An association of the protein has been reported with a neuron-specific kinesin (KIF1A) involved in transport of synaptic vesicle precursors in the axon, leading to the suggestion that it may function in vesicular transport (Koshizuka et al., 2005). These features prompted a comparison (Koshizuka et al., 2005) with membrane protein US9, which is a tail-anchored, type 2 membrane protein involved in transport of virus proteins towards the axon terminus, probably in vesicles (Brideau et al., 1998; Lyman et al., 2007). An interaction detected between membrane protein UL56 and myristylated tegument protein (encoded by gene UL49A in HHV2; alternatively named UL49.5), and colocalization of the complex to the Golgi apparatus and aggresome-like structures, suggests that it may have a role in virus maturation and egress (Koshizuka et al., 2006). Membrane protein UL56 also interacts with the ubiquitin ligase Nedd4 via its PPXY motifs and promotes its ubiquitination (Ushijima et al., 2008).

Some functional investigations have been carried out on other orthologues. Deletion of the EHV1 or SuHV1 gene has no effect on growth in cell culture (Sun and Brown, 1994; Baumeister et al., 1995), whereas the VZV protein is required for efficient growth of virus in cell culture and in an animal model system (Zhang et al., 2007).

### 4.3 Gene US8A

**Fig. 2:**
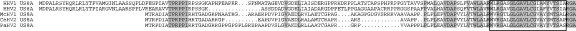
Amino acid sequence alignment of protein US8A from the genus *Simplexvirus*. Conserved residues are highlighted in grey, and predicted transmembrane domains are boxed.

HHV1 gene US8A (alternatively named US8.5) appeared late on the scene. It was overlooked in the genome sequence analyses (McGeoch et al., 1985, 1988), and was discovered later by Georgopoulou et al. (1993). In a comparative description of the HHV2 gene content, Dolan et al. (1998) evaluated US8A as probably specific to the genus *Simplexviru*s, and noted a positional counterpart in EHV1, a member of the genus *Varicellovirus*. Georgopoulou et al. (1993) characterized an internally tagged form of the HHV1 protein as locating to nucleoli. However, as registered by Dolan et al. (1998), the sequence used in these experiments was frameshifted, so the 16 residues at the C terminus of the protein would have been replaced. This raises a question as to whether the mutated protein might have localized inappropriately. A comparative approach (Fig. 2) indicates that the US8A protein has the sequence properties of a tail-anchored, type 2 membrane protein (N terminus in the cytoplasm). It is notable that the adjacent gene (US9) also encodes a tail-anchored, type 2 membrane protein, though it is unrelated in sequence to membrane protein US8A. A gene that is positionally equivalent to gene US8A is present in members of other genera in the subfamily *Alphaherpesvirinae*, and the same protein name (membrane protein US8A) is currently assigned (Table 2).

### 4.4 UL30A gene family

**Fig. 3:**
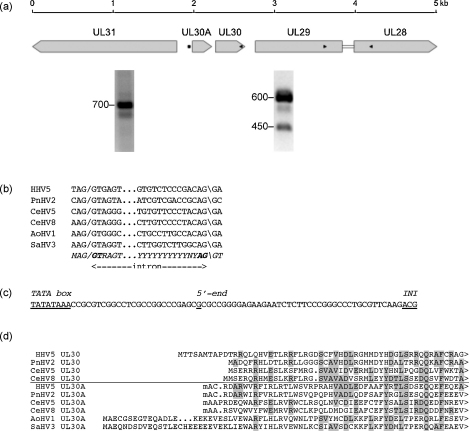
Splicing of UL28 and UL29 and identification of the UL30 gene family in the genus *Cytomegalovirus*. (a) Layout of ORFs in the relevant region of the HHV5 genome (inverted from its standard depiction), with images of ethidium bromide-stained agarose gels showing RT-PCR and 5′-RACE products (sizes in bp) from total cell RNA harvested late during infection of human fibroblast cells by HHV5 strain Merlin. The RT-PCR products in the right panel relate to the region spanning UL28 and UL29 (primers 5-GTCGCCCAGCATGATGCCGTGCAG-3′ and 5′-CTTTCACCGCGTGCGGATTCTCTG-3′; black arrowheads). The 5′-RACE products in the left panel relate to the region upstream from UL30A (primer 5′-TCTACGGAGACCTGACAGCAGTTG-3′; black arrowhead). The mRNA 5′-end upstream from UL30 is marked with a black square (see panel (c) for details. (b) Nucleotide sequence alignment of predicted (confirmed in the case of HHV5) splice donor (/) and acceptor (\) sites flanking the intron linking UL28 and UL29. The italicized line shows the canonical sequences, with critical residues in bold type. (c) The proposed TATA box, mapped mRNA 5′-end and putative non-ATG (ACG) translational initiation codon (*INI*) for UL30A in HHV5 strain Merlin. The same 5′-end was mapped experimentally for HHV5 strain AD169. (d) Predicted amino acid sequence alignment of the N-terminal portion of the UL30A and UL30 proteins. The lower case residue at the start of the UL30A sequences indicates the proposed encoding of an initiating methionine residue by the non-ATG codon. Residues that are conserved respectively in at least two UL30A sequences and at least one UL30 sequence (or vice versa) are shaded. The C-termini of the proteins are indicated by >.

HHV5 and its closest relative (PnHV2) are members of the genus *Cytomegalovirus* in the subfamily *Betaherpesvirinae*. The UL28 coding region in these viruses was predicted several years ago to be spliced to an unidentified upstream exon (Davison et al., 2003). The layout of UL28 in relation to genes upstream is shown in Fig. 3a. A subsequent comparative analysis involving members or potential members of the genus *Cytomegalovirus* from Old and New World primates (HHV5, PnHV2, CeHV5 and CeHV8 for the former; AoHV1 and SaHV3 for the latter) strongly suggested that the upstream exon is UL29 (Fig. 3b). Reverse-transcription PCR (RT-PCR) confirmed the expression of the predicted spliced HHV5 RNA (as represented by the 450 bp RT-PCR product in Fig. 3a), as well as the unspliced RNA (as represented by the 600 bp product). The appropriate amendment to the HHV5 RefSeq was made early in 2009. The intron has also been detected independently (Mitchell et al., 2009). Thus, two previous coding regions (UL28 and UL29) present in primate members of the genus *Cytomegalovirus* have been conflated to a single gene (UL29).

During transcript mapping experiments, we mapped the major 5′-end upstream from HHV5 UL29 to a nucleotide position approximately 800 bp upstream from the translational initiation codon, and 300 bp upstream from the UL30 translational initiation codon (as represented by the 700 bp 5′-RACE product in Fig. 3a). This transcriptional initiation site is located an appropriate distance downstream from a candidate TATA box (Fig. 3c). Comparative analysis then led to the discovery of a potential coding region (UL30A) located in the 300 bp region between the transcriptional initiation site and the UL30 translational initiation codon. This region potentially encodes a protein that is conserved in other Old World primate members of the genus *Cytomegalovirus* and is related to protein UL30 (Fig. 3d). However, none of the UL30A coding regions in these viruses possesses an appropriately positioned translational initiation codon (ATG). It is possible that translational initiation occurs from a non-ATG codon, in this case ACG, which has been shown to function as an initiation codon in eukaryotic systems (Anderson and Buzash-Pollert, 1985; Peabody, 1989) and viruses such as adeno-associated virus (Becerra et al., 1988) and Sendai virus (Curran and Kolakofsky, 1988). Indeed, a few respectable herpesvirus coding regions lack an ATG initiation codon and have been proposed to utilise a non-ATG codon; for example, that encoding HHV2 tegument protein UL16 (Dolan et al., 1998). The New World primate viruses that potentially belong to the genus *Cytomegalovirus* appear to have UL30A orthologues with ATG initiation codons, but lack UL30 orthologues (Fig. 3d). These findings indicate the presence of paralogues (UL30 and UL30A) that together constitute the novel UL30 gene family.

## Conflict of interest

The author has no conflict of interest.

## Figures and Tables

**Table 1:** Herpesvirus classification and genome sequences.

Taxon name^a^	Common name^b^	Acronym^c^	Strain name^d^	GenBank accession	RefSeq accession	Genome size (kb)^e^	Reference
**Order*****Herpesvirales***							
**Family*****Herpesviridae***							
**Subfamily*****Alphaherpesvirinae***							

**Genus*****Iltovirus***							
Gallid herpesvirus 1*	Infectious laryngotracheitis virus	GaHV1	Composite of 6 strains		NC_006623	148,687	Thureen and Keeler (2006)
*Psittacid herpesvirus 1*	Pacheco's disease virus	PsHV1	97-0001	AY372243	NC_005264	163,025	Thureen and Keeler (2006)

**Genus*****Mardivirus***							
*Columbid herpesvirus 1*	Pigeon herpesvirus	CoHV1	KP21/23				
Gallid herpesvirus 2*	Marek's disease virus type 1	GaHV2	Md5	AF243438	NC_002229	177,874	Tulman et al. (2000)
			GA	AF147806		174,077	Lee et al. (2000)
			CU-2	EU499381		176,922	Spatz and Rue (2008)
			Md11 [BAC]	AY510475			Niikura et al. (2006)
			CVI988 [BAC]	DQ530348			Spatz et al. (2007a)
			RB-1B [BAC]	EF523390			Spatz et al. (2007b)
			584A [BAC]	EU627065			Spatz et al. (2008)
*Gallid herpesvirus 3*	Marek's disease virus type 2	GaHV3	HPRS24	AB049735	NC_002577	164,270	Izumiya et al. (2001)
*Meleagrid herpesvirus 1*	Turkey herpesvirus	MeHV1	FC126 (Burmester)	AF291866	NC_002641	159,160	Afonso et al. (2001)
			FC126	AF282130		161,484^f^	Kingham et al. (2001)

**Genus*****Simplexvirus***							
*Ateline herpesvirus 1*	Spider monkey herpesvirus	AtHV1					
*Bovine herpesvirus 2*	Bovine mammillitis virus	BoHV2					
*Cercopithecine herpesvirus 2*	Simian agent 8	CeHV2	B264	AY714813	NC_006560	150,715	Tyler et al. (2005)
Human herpesvirus 1*	Herpes simplex virus type 1	HHV1	17	X14112	NC_001806	152,261	McGeoch et al. (1988)
			17 [BAC]	FJ593289			Cunningham and Davison (unpublished)
*Human herpesvirus 2*	Herpes simplex virus type 2	HHV2	HG52	Z86099	NC_001798	154,746	Dolan et al. (1998)
*Leporid herpesvirus 4*	Leporid herpesvirus 4	LeHV4					
*Macacine herpesvirus 1*	B virus	McHV1	E2490	AF533768	NC_004812	156,789	Perelygina et al. (2003)
*Macropodid herpesvirus 1*	Parma wallaby herpesvirus	MaHV1					
*Macropodid herpesvirus 2*	Dorcopsis wallaby herpesvirus	MaHV2					
*Papiine herpesvirus 2*	Herpesvirus papio 2	PaHV2	X313	DQ149153	NC_007653	156,487	Tyler and Severini (2006)
*Saimiriine herpesvirus 1*	Marmoset herpesvirus	SaHV1					

**Genus*****Varicellovirus***							
*Bovine herpesvirus 1*	Infectious bovine rhinotracheitis virus	BoHV1	Composite of 5 strains	AJ004801	NC_001847	135,301	Schwyzer and Ackermann (1996)
*Bovine herpesvirus 5*	Bovine encephalitis herpesvirus	BoHV5	SSV507/99	AY261359	NC_005261	137,821	Delhon et al. (2003)
*Bubaline herpesvirus 1*	Water buffalo herpesvirus	BuHV1					
*Canid herpesvirus 1*	Canine herpesvirus	CaHV1					
*Caprine herpesvirus 1*	Goat herpesvirus	CpHV1					
*Cercopithecine herpesvirus 9*	Simian varicella virus	CeHV9	Delta	AF275348	NC_002686	124,784	Gray et al. (2001)
*Cervid herpesvirus 1*	Red deer herpesvirus	CvHV1					
*Cervid herpesvirus 2*	Reindeer herpesvirus	CvHV2					
*Equid herpesvirus 1*	Equine abortion virus	EHV1	Ab4	AY665713	NC_001491	150,224	Telford et al. (1992)
			V592	AY464052		149,430	Nugent et al. (2006)
*Equid herpesvirus 3*	Equine coital exanthema virus	EHV3					
*Equid herpesvirus 4*	Equine rhinopneumonitis virus	EHV4	NS80567	AF030027	NC_001844	145,597	Telford et al. (1998)
*Equid herpesvirus 9*	Gazelle herpesvirus	EHV9	P19	AP010838	NC_011644	148,371	Fukushi et al. (unpublished)
*Felid herpesvirus 1*	Feline herpesvirus 1	FHV1	C-27 [BAC]	FJ478159	NC_013590		Tai et al. (unpublished)
Human herpesvirus 3*	Varicella-zoster virus	HHV3	Dumas	X04370	NC_001348	124,884	Davison and Scott (1986)
			Oka vaccine	AB097932		125,078	Gomi et al. (2002)
			Oka parental	AB097933		125,125	Gomi et al. (2002)
			MSP	AY548170		124,883	Grose et al. (2004)
			BC	AY548171		125,459	Grose et al. (2004)
			SD	DQ479953		125,087	Peters et al. (2006)
			Kel	DQ479954		125,374	Peters et al. (2006)
			11	DQ479955		125,370	Peters et al. (2006)
			22	DQ479956		124,868	Peters et al. (2006)
			03-500	DQ479957		125,239	Peters et al. (2006)
			36	DQ479958		125,030	Peters et al. (2006)
			49	DQ479959		125,041	Peters et al. (2006)
			8	DQ479960		125,451	Peters et al. (2006)
			32 passage 5	DQ479961		124,945	Peters et al. (2006)
			32 passage 22	DQ479962		125,084	Peters et al. (2006)
			32 passage 72	DQ479963		125,169	Peters et al. (2006)
			DR	DQ452050		124,770	Norberg et al. (2006)
			CA123	DQ457052		124,771	Norberg et al. (2006)
			Oka Varilrix	DQ008354		124,821	Tillieux et al. (2008)
			Oka Varivax	DQ008355		124,815	Tillieux et al. (2008)
			NH293	DQ674250		124,811	Loparev et al. (2009)
			SVETA	EU154348		124,813	Loparev et al. (2009)
			HJ0	AJ871403		124,928	Fickenscher et al. (unpublished)
*Phocid herpesvirus 1*	Harbour seal herpesvirus	PhoHV1					
*Suid herpesvirus 1*	Pseudorabies virus	SuHV1	Composite of 6 strains	BK001744	NC_006151	143,461	Klupp et al. (2004)

**Unassigned species in the subfamily**							
*Chelonid herpesvirus 5*	Chelonid fibropapilloma-associated herpesvirus	ChHV5					
*Chelonid herpesvirus 6*	Lung-eye-trachea disease-associated virus	ChHV6					

**Subfamily*****Betaherpesvirinae***							
**Genus*****Cytomegalovirus***							
*Cercopithecine herpesvirus 5*	Simian cytomegalovirus	CeHV5	2715	FJ483968	NC_012783	226,204	Dolan et al. (unpublished)
			Colburn	FJ483969		219,524	Dolan et al. (unpublished)
Human herpesvirus 5*	Human cytomegalovirus	HHV5	Merlin	AY446894	NC_006273	235,646	Dolan et al. (2004)
			AD169 varUK	BK000394		230,290	Chee et al. (1990)
			AD169 varUC	FJ527563		231,781	Bradley et al. (2009)
			Towne varATCC	FJ616285		235,147	Bradley et al. (2009)
			3157	GQ221974		235,154	Cunningham et al. (2010)
			3301	GQ466044		235,694	Cunningham et al. (2010)
			HAN13	GQ221973		236,219	Cunningham et al. (2010)
			HAN20	GQ396663		235,728	Cunningham et al. (2010)
			HAN38	GQ396662		236,112	Cunningham et al. (2010)
			JP	GQ221975		236,375	Cunningham et al. (2010)
			Towne varS [BAC]	AY315197			Dunn et al. (2003)
			AD169 varATCC [BAC]	AC146999			Murphy et al. (2003)
			FIX [BAC]	AC146907			Murphy et al. (2003)
			PH [BAC]	AC146904			Murphy et al. (2003)
			Towne varS [BAC]	AC146851			Murphy et al. (2003)
			Toledo [BAC]	AC146905			Murphy et al. (2003)
			TR [BAC]	AC146906			Murphy et al. (2003)
			TB40/E [BAC]	EF999921			Sinzger et al. (2008)
			Towne varL [BAC]	GQ121041			Cui et al. (unpublished)
			Merlin [BAC]	GU179001			Stanton et al. (unpublished)
*Macacine herpesvirus 3*	Rhesus cytomegalovirus	McHV3	68-1	AY186194	NC_006150	221,454	Hansen et al. (2003)
			180.92	DQ120516		215,678	Rivailler et al. (2006)
*Panine herpesvirus 2*	Chimpanzee cytomegalovirus	PnHV2	Heberling	AF480884	NC_003521	241,087	Davison et al. (2003)

**Unassigned viruses in the genus**							
Aotine herpesvirus 1	Herpesvirus aotus type 1	AoHV1	S 34E	FJ483970		219,474	Dolan et al. (unpublished)
Aotine herpesvirus 3^g^	Herpesvirus aotus type 3	AoHV3					
Saimiriine herpesvirus 3	Squirrel monkey cytomegalovirus	SaHV3	SqSHV	FJ483967		[189,956]	Dolan et al. (unpublished)

**Genus*****Muromegalovirus***							
Murid herpesvirus 1*	Murine cytomegalovirus	MuHV1	Smith	U68299	NC_004065	230,278	Rawlinson et al. (1996)
			C4A	EU579861		230,111	Smith et al. (2008)
			G4	EU579859		230,227	Smith et al. (2008)
			WP15B	EU579860		230,118	Smith et al. (2008)
			K181 [BAC]	AM886412			Smith et al. (2008)
*Murid herpesvirus 2*	Rat cytomegalovirus	MuHV2	Maastricht	AF232689	NC_002512	230,138	Vink et al. (2000)

**Genus*****Roseolovirus***							
*Human herpesvirus 6*	Human herpesvirus 6	HHV6	U1102	X83413	NC_001664	159,322	Gompels et al. (1995)
			Z29	AF157706	NC_000898	162,114	Dominguez et al. (1999)
			HST	AB021506		161,573	Isegawa et al. (1999)
*Human herpesvirus 7*	Human herpesvirus 7	HHV7	RK	AF037218	NC_001716	153,080	Megaw et al. (1998)
			JI	U43400		144,861	Nicholas (1996)

**Genus*****Probiscivirus***							
*Elephantid herpesvirus 1*	Elephant endotheliotropic herpesvirus	ElHV1					

**Unassigned species in the subfamily**							
*Caviid herpesvirus 2*	Guinea pig cytomegalovirus	CavHV2	21222 [BAC]	FJ355434	NC_011587		Schleiss et al. (2008)
*Suid herpesvirus 2*	Pig cytomegalovirus	SuHV2					
*Tupaiid herpesvirus 1*	Tupaiid herpesvirus	TuHV1	2	AF281817	NC_002794	195,859	Bahr and Darai (2001)

**Subfamily*****Gammaherpesvirinae***							
**Genus*****Lymphocryptovirus***							
*Callitrichine herpesvirus 3*	Marmoset lymphocryptovirus	CalHV3	CJ0149	AF319782	NC_004367	149,696	Rivailler et al. (2002a)
*Cercopithecine herpesvirus 14*	African green monkey EBV-like virus	CeHV14					
*Gorilline herpesvirus 1*	Gorilla herpesvirus	GoHV1					
Human herpesvirus 4*	Epstein–Barr virus	HHV4	B95-8/Raji	AJ507799	NC_007605	171,823	Baer et al. (1984) and de Jesus et al. (2003)
			GD1	AY961628		171,657	Zeng et al. (2005)
			AG876	DQ279927		172,764	Dolan et al. (2006)
*Macacine herpesvirus 4*	Rhesus lymphocryptovirus	McHV4	LCL8664	AY037858	NC_006146	171,096	Rivailler et al. (2002b)
*Panine herpesvirus 1*	Herpesvirus pan	PnHV1					
*Papiine herpesvirus 1*	Herpesvirus papio	PaHV1					
*Pongine herpesvirus 2*	Orangutan herpesvirus	PoHV2					

**Genus*****Macavirus***							
Alcelaphine herpesvirus 1*	Wildebeest-associated malignant catarrhal fever virus	AlHV1	C500	AF005370	NC_002531	130,608	Ensser et al. (1997)
				AF005368		1113	
*Alcelaphine herpesvirus 2*	Hartebeest malignant catarrhal fever virus	AlHV2					
*Bovine herpesvirus 6*	Bovine lymphotropic herpesvirus	BoHV6					
*Caprine herpesvirus 2*	Caprine herpesvirus 2	CpHV2					
*Hippotragine herpesvirus 1*	Roan antelope herpesvirus	HiHV1					
*Ovine herpesvirus 2*	Sheep-associated malignant catarrhal fever virus	OvHV2	BJ1035	AY839756	NC_007646	135,135	Hart et al. (2007)
			Composite of several strains	DQ198083		[131,621]	Taus et al. (2007)
*Suid herpesvirus 3*	Porcine lymphotropic herpesvirus 1	SuHV3					
*Suid herpesvirus 4*	Porcine lymphotropic herpesvirus 2	SuHV4					
*Suid herpesvirus 5*	Porcine lymphotropic herpesvirus 3	SuHV5					

**Genus*****Percavirus***							
Equid herpesvirus 2*	Equine herpesvirus 2	EHV2	86/67	U20824	NC_001650	184,427	Telford et al. (1995)
*Equid herpesvirus 5*	Equine herpesvirus 5	EHV5					
*Mustelid herpesvirus 1*	Badger herpesvirus	MusHV1					

**Genus*****Rhadinovirus***							
*Ateline herpesvirus 2*	Herpesvirus ateles strain 810	AtHV2					
*Ateline herpesvirus 3*	Herpesvirus ateles	AtHV3	73	AF083424	NC_001987	108,409	Albrecht (2000)
				AF126541		1582	
*Bovine herpesvirus 4*	Bovine herpesvirus 4	BoHV4	66-p-347	AF318573	NC_002665	108,873	Zimmermann et al. (2001)
				AF092919		2267	
*Human herpesvirus 8*	Human herpesvirus 8	HHV8	GK18	AF148805	NC_009333	137,969	Rezaee et al. (2006)
			BC-1	U75698		[137,508]	Russo et al. (1996)
				U75699		801	
			Composite of 2 strains	U93872		[133,661]	Neipel et al. (1997)
			219 [BAC]	GQ994935			Brulois et al. (unpublished)
*Macacine herpesvirus 5*	Rhesus rhadinovirus	McHV5	17577	AF083501	NC_003401	133,719	Searles et al. (1999)
			26-95	AF210726		130,733	Alexander et al. (2000)
				AY528864		131,217	Hansen et al. (unpublished)
*Murid herpesvirus 4*	Murine herpesvirus 68	MuHV4	g2.4 (WUMS)	U97553	NC_001826	119,450	Virgin et al. (1997)
			g2.4	AF105037		119,550	Nash et al. (2001)
Saimiriine herpesvirus 2*	Herpesvirus saimiri	SaHV2	A11	X64346	NC_001350	112,930	Albrecht et al. (1992)
				K03361		1444	
			C488	AJ410493		113,027	Ensser et al. (2003)
				AJ410494		1458	

**Unassigned viruses in the genus**							
Leporid herpesvirus 1	Cottontail rabbit herpesvirus	LeHV1					
Leporid herpesvirus 2	Herpesvirus cuniculi	LeHV2					
Leporid herpesvirus 3	Herpesvirus sylvilagus	LeHV3					
Marmodid herpesvirus 1	Woodchuck herpesvirus	MarHV1					

**Unassigned species in the subfamily**							
*Equid herpesvirus 7*	Asinine herpesvirus 2	EHV7					
*Phocid herpesvirus 2*	Phocid herpesvirus 2	PhoHV2					
*Saguinine herpesvirus 1*	Herpesvirus saguinus	SgHV1					

**Unassigned species in the family**							
*Iguanid herpesvirus 2*	Iguana herpesvirus	IgHV2					

**Unassigned viruses in the family**							
Acciptrid herpesvirus 1	Bald eagle herpesvirus	AcHV1					
Anatid herpesvirus 1	Duck enteritis virus	AnHV1	VAC	EU082088	NC_013036	158,091	Li et al. (2009)
Boid herpesvirus 1	Boa herpesvirus	BoiHV1					
Callitrichine herpesvirus 2	Marmoset cytomegalovirus	CalHV2					
Caviid herpesvirus 1	Guinea pig herpesvirus	CavHV1					
Caviid herpesvirus 3	Guinea pig herpesvirus 3	CavHV3					
Cebine herpesvirus 1	Capuchin herpesvirus AL-5	CbHV1					
Cebine herpesvirus 2	Capuchin herpesvirus AP-18	CbHV2					
Cercopithecine herpesvirus 3^h^	SA6	CeHV3					
Cercopithecine herpesvirus 4	SA15	CeHV4					
Chelonid herpesvirus 1	Grey patch disease-associated virus	ChHV1					
Chelonid herpesvirus 2	Pacific pond turtle herpesvirus	ChHV2					
Chelonid herpesvirus 3	Painted turtle herpesvirus	ChHV3					
Chelonid herpesvirus 4	Argentine turtle herpesvirus	ChHV4					
Ciconiid herpesvirus 1	Black stork herpesvirus	CiHV1					
Cricetid herpesvirus	Hamster herpesvirus	CrHV1					
Elapid herpesvirus 1	Indian cobra herpesvirus	EpHV1					
Erinaceid herpesvirus 1	European hedgehog herpesvirus	ErHV1					
Falconid herpesvirus 1^i^	Falcon inclusion body disease virus	FaHV1					
Gruid herpesvirus 1	Crane herpesvirus	GrHV1					
Iguanid herpesvirus 1	Green iguana herpesvirus	IgHV1					
Lacertid herpesvirus	Green lizard herpesvirus	LaHV1					
Macacine herpesvirus 6	Rhesus leukocyte-associated herpesvirus strain 1	McHV6					
Macacine herpesvirus 7	Herpesvirus cyclopis	McHV7					
Murid herpesvirus 3	Mouse thymic herpesvirus	MuHV3					
Murid herpesvirus 5	Field mouse herpesvirus	MuHV5					
Murid herpesvirus 6	Sand rat nuclear inclusion agent	MuHV6					
Murid herpesvirus 7	Wood mouse herpesvirus	MuHV7	WM8	GQ169129		120108	Hughes et al. (2010)
Ovine herpesvirus 1	Sheep pulmonary adenomatosis-associated herpesvirus	OvHV1					
Perdicid herpesvirus 1	Bobwhite quail herpesvirus	PdHV1					
Phalacrocoracid herpesvirus 1	Cormorant herpesvirus	PhHV1					
Procyonid herpesvirus 1	Kinkajou herpesvirus	PrHV1					
Sciurid herpesvirus 1	Ground squirrel cytomegalovirus	ScHV1					
Sciurid herpesvirus 2	Ground squirrel herpesvirus	ScHV2					
Sphenicid herpesvirus 1	Black footed penguin herpesvirus	SpHV1					
Strigid herpesvirus 1^i^	Owl hepatosplenitis virus	StHV1					

**Family*****Alloherpesviridae***							
**Genus*****Batrachovirus***							
Ranid herpesvirus 1*	Lucké tumor herpesvirus	RaHV1	McKinnell	DQ665917	NC_008211	220,859	Davison et al. (2006)
*Ranid herpesvirus 2*	Frog virus 4	RaHV2	Rafferty	DQ665652	NC_008210	231,801	Davison et al. (2006)

**Genus*****Cyprinivirus***							
*Cyprinid herpesvirus 1*	Carp pox herpesvirus	CyHV1					
*Cyprinid herpesvirus 2*	Goldfish haematopoietic necrosis virus	CyHV2					
Cyprinid herpesvirus 3*	Koi herpesvirus	CyHV3	U	DQ657948	NC_009127	295,146	Aoki et al. (2007)
			J	AP008984		295,052	Aoki et al. (2007)
			I	DQ177346		295,138	Aoki et al. (2007)

**Genus*****Ictalurivirus***							
Ictalurid herpesvirus 1*	Channel catfish virus	IcHV1	Auburn 1	M75136	NC_001493	134,226	Davison (1992)
*Ictalurid herpesvirus 2*	Black bullhead herpesvirus	IcHV2					
*Acipenserid herpesvirus 2*	White sturgeon herpesvirus 2	AciHV2					

**Genus*****Salmonivirus***							
Salmonid herpesvirus 1*	Herpesvirus salmonis	SalHV1					
*Salmonid herpesvirus 2*	Oncorhynchus masou herpesvirus	SalHV2					
*Salmonid herpesvirus 3*	Epizootic epitheliotropic disease virus	SalHV3					

**Unassigned viruses in the family**							
Acipenserid herpesvirus 1	White sturgeon herpesvirus 1	AciHV1					
Anguillid herpesvirus 1	Eel herpesvirus	AngHV1	500138	FJ940765	NC_013668	248,531	van Beurden et al. (2010)
Esocid herpesvirus 1	Northern pike herpesvirus	EsHV1					
Percid herpesvirus 1	Walleye epidermal hyperplasia herpesvirus	PeHV1					
Pleuronectid herpesvirus 1	Turbot herpesvirus	PlHV1					

**Family*****Malacoherpesviridae***							
**Genus*****Ostreavirus***							
Ostreid herpesvirus 1*	Oyster herpesvirus	OsHV1		AY509253	NC_005881	207,439	Davison et al. (2005b)

a The type species in each genus is indicated by an asterisk. Formal taxonomic names are italicized. The names of tentative species and unassigned viruses have no taxonomic standing are not italicized.

b Virus common names have no taxonomic standing. Some viruses have more than one common name. A single, English, common name is given for each virus.

c Acronyms or abbreviations may apply to viruses, not species, and have no taxonomic standing. A hyphen may precede the number.

d Where a sequence was determined from a bacterial artificial chromosome (BAC), this is indicated in square brackets.

e The sizes of complete or almost complete virus genomes are shown; the latter in square brackets. The sizes of BAC-derived sequences are not shown, since they may contain all or part of the sequence of the BAC vector. Sizes in square brackets indicate sequences that are incomplete in extent or construction. For members of the subfamily *Gammaherpesvirinae* other than EHV2, actual genome sizes are larger than those listed (150–180 kbp), owing to the presence of variable copy numbers of the terminal repeat flanking the unique region. A single size value represents either the unique region only or the unique region flanked by partial terminal repeats. Two values represent the unique region and the terminal repeat.

f The size is that of the genome sequence reconstructed from the GenBank accession.

g This is probably a strain of AoHV1 (Ebeling et al., 1983).

h This is a strain of CeHV5 (G. Hayward, personal communication).

i This is a strain of CoHV1 (Gailbreath and Oaks, 2008).

**Table 2:** Names and conservation of proteins in members of the subfamily *Alphaherpesvirinae*.





^a^The viruses are grouped taxonomically, as indicated by the vertical lines, though AnHV1 has not yet been classified. The presence () or absence () of apparent gene orthologues is indicated for each genome, with the U_L_ and U_S_ regions oriented to correspond to the conventional arrangement in the varicelloviruses. Gene names differ between viruses, and are available in the RefSeqs. ^b^The names of proteins encoded by genes in the inverted repeats in certain viruses, and which are therefore listed twice, are italicized. ^c^Core genes, inherited from an ancestor of alpha-, beta- and gammaherpesviruses; alpha genes, inherited from an ancestor of alphaherpesviruses. This is a definition based on evolution – certain core or alpha genes may have been lost in some lineages. ^d^Genes that have paralogues in the same or other herpesviruses belong to gene families. The paralogues have presumably arisen by gene duplication.
